# Public Parks and Wellbeing in Urban Areas of the United States

**DOI:** 10.1371/journal.pone.0153211

**Published:** 2016-04-07

**Authors:** Lincoln R. Larson, Viniece Jennings, Scott A. Cloutier

**Affiliations:** 1 Department of Parks, Recreation, & Tourism Management, Clemson University, Clemson, South Carolina, United States of America; 2 USDA Forest Service, Southern Research Station, Integrating Human and Natural Systems, Athens, Georgia, United States of America; 3 Julie Ann Wrigley Global Institute of Sustainability, Arizona State University, Tempe, Arizona, United States of America; Auburn University, UNITED STATES

## Abstract

Sustainable development efforts in urban areas often focus on understanding and managing factors that influence all aspects of health and wellbeing. Research has shown that public parks and green space provide a variety of physical, psychological, and social benefits to urban residents, but few studies have examined the influence of parks on comprehensive measures of subjective wellbeing at the city level. Using 2014 data from 44 U.S. cities, we evaluated the relationship between urban park quantity, quality, and accessibility and aggregate self-reported scores on the Gallup-Healthways Wellbeing Index (WBI), which considers five different domains of wellbeing (e.g., physical, community, social, financial, and purpose). In addition to park-related variables, our best-fitting OLS regression models selected using an information theory approach controlled for a variety of other typical geographic and socio-demographic correlates of wellbeing. Park quantity (measured as the percentage of city area covered by public parks) was among the strongest predictors of overall wellbeing, and the strength of this relationship appeared to be driven by parks’ contributions to physical and community wellbeing. Park quality (measured as per capita spending on parks) and accessibility (measured as the overall percentage of a city’s population within ½ mile of parks) were also positively associated with wellbeing, though these relationships were not significant. Results suggest that expansive park networks are linked to multiple aspects of health and wellbeing in cities and positively impact urban quality of life.

## Introduction

Over 50% of the global population currently resides in urban areas, and that proportion continues to grow rapidly [1]. In the United States, four out of five people currently live in cities [2]. The urbanization of human society has important implications for health and well-being [3, 4]. On one hand, dense urban populations may have more access to health care and amenities that promote healthy lifestyles. On the other hand, urban environments cultivate a variety of environmental (e.g., pollution, sanitation concerns) and social (e.g., segregation, socioeconomic disparities) stressors that make them more susceptible to health problems [5]. Understanding, quantifying, and managing the variables that influence all aspects of human welfare has become a major challenge in the movement to build sustainable, healthy cities.

To achieve this goal, city planners and managers must determine how to efficiently and effectively monitor and manage for health outcomes. Historically, standards have been based on metrics of objective population health such as morbidity and mortality rates [3]. Although such measures are undoubtedly important, they often fail to capture more dynamic aspects of human welfare. Consequently, many researchers and practitioners across multiple disciplines are turning toward quality of life measures, including life satisfaction, happiness, and wellbeing as comprehensive strategies for assessing holistic health outcomes [6–9]. A key question has therefore become: what are the social and environmental determinants of human wellbeing in urban areas?

### Wellbeing as a Comprehensive Measure of Health

Scientists have historically measured wellbeing using objective indicators (e.g., GDP, health, employment, literacy, poverty), but such coarse metrics fail to acknowledge all of the parameters, both positive and negative, that influence a person’s evaluation of life [10]. The concept of subjective wellbeing more explicitly considers how people evaluate multiple aspects of their day-to-day life [11]. Modern measures of wellbeing that account for cognitive evaluations (i.e., evaluative wellbeing) and reactions to experiences (i.e., experienced wellbeing) have therefore become the “currency of a life that matters” [12]. Such perspectives suggest that wellbeing is ultimately a social and public good, not just a private or individual concern. Discussions surrounding wellbeing therefore provide a “powerful invitation to discuss and assess how society facilitates or inhibits the enjoyment of good lives” [13] (p. 33). Consequently, many countries around the world are now measuring happiness (a term often used interchangeably with life satisfaction and subjective wellbeing) as a national standard [14], and some cities within the U.S. have also made residential happiness a top priority [15]. As wellbeing continues to gain traction in the public health arena, more urban policy-makers and planners are likely to respond [16, 17]. These trends underscore the growing importance of ongoing efforts to identify the factors that create happy, healthy cities [15, 18]. As conceptualization of wellbeing evolves, more indices are beginning to include core elements (e.g. physical health, mental health, community attachment, and economic security) that illustrate the interdependency among various domains of health and happiness [8, 19]. Research is also beginning to identify some of the key correlates that may help to generate positive wellbeing outcomes.

### Factors Associated with Wellbeing

A substantial body of literature has also explored the relationship between socio-economic standing and wellbeing. While higher income is generally assumed to be associated with greater wellbeing, the relationship may be mediated by other factors [6, 7]. For example, in her analysis of the paradox of “happy peasants and miserable millionaires,” Graham [20] noted that income alone was a poor predictor of happiness, highlighting the need for additional considerations such as social and economic context. Kahneman & Deaton [21] reported similar variability, and noted that income was associated with life satisfaction, but not emotional wellbeing. Education, which is often used as a proxy for human capital, has also emerged as an important correlate of wellbeing. Higher levels of education are typically linked to higher levels of self-reported wellbeing [22–24], though this pattern may not hold under all circumstances. For instance, Diener et al. [6] described the phenomenon of diminishing returns, whereby higher education and income leads to increasing pressure regarding goals and aspirations and, hence, more perceived challenges and dissatisfaction in life. Other measures associated with economic prosperity such as the availability of jobs and employment opportunities are also considered to be key wellbeing correlates [18, 24]; in most studies, higher levels of unemployment are generally associated with lower levels of happiness [22, 24, 25]. Collectively, evidence suggests that all of these socio-economic indicators should be included in models predicting wellbeing, regardless of effect size or direction.

Wellbeing, of course, is not strictly driven by socio-economic factors. Strength of social relationships, for example, can drastically influence how a person evaluates his/her life [26, 27]. However, the extent and intensity of such relationships can be difficult to measure–particularly at the city level. At these larger scales, broader measures of population densities and trajectories (growth and decay) may be useful indicators of residents’ wellbeing. Although some research suggests population density may have minimal effects on wellbeing within cities [22], it appears to be relevant at the state level, with residents of less dense states typically reporting higher levels of happiness [24]. Safety, educational opportunities, and access to arts and culture can impact social and human capital, which are considered powerful drivers of urban well-being [18, 22]. Other features of urban infrastructure (e.g. roads, transportation, social gathering spaces) affect commuting time and connectivity, all of which are linked to happiness [18, 24, 28, 29]. Studies also suggest that environmental factors such as climate and geography [30–32], as well as indicators of ecological health such as air and water quality and sustainability-oriented policies and practices, are also linked to urban wellbeing [15, 33]. Collectively, these relationships demonstrate how carefully planned urban development can offer psychological enrichment, promote physical health, and foster a greater sense of community as people become attached to their life-places [34, 35]. Green spaces such as public parks are a valuable urban feature that supports all of these goals.

### Urban Parks, Green Space and Wellbeing

A substantial body of research articulates the multiple contributions of urban green space, a category of land cover that includes public parks and other (public or private) vegetated areas, to human health and wellbeing [4, 36–42]. These green spaces provide ecosystem services that support human welfare in multiple ways [43–45]. For example, studies of specific neighborhoods and cities imply that proximity to and use of urban green space is positively associated with the physical activity levels and cardiovascular health of urban residents [46–49]. Parks and green space also support vegetation that contributes to other aspects of physical health by reducing heat effects [50], regulating air and water pollution [51], and enhancing access to nutritious fruits and vegetables [52, 53].

Although research focused on the physical health benefits of urban greenspace has received the most dedicated coverage [41], parks and natural areas also impact the psychological health of city residents [4, 9, 41, 54]. For example, individuals living in greener urban areas display more positive indicators of mental health than those who live in less green settings [55], including fewer symptoms of depression [56], and lower levels of self-reported [57] and biologically-measured stress [58]. Greener areas are also associated with cognitive development and learning outcomes [59–61]. Moreover, green spaces may facilitate social connections, neighborhood satisfaction and community attachment in many urban settings [62–66]. All of these findings suggest that cities with higher concentrations of parks and other green spaces provide greater opportunities for happiness than their park-barren counterparts [67].

Although findings suggest parks and green space are key correlates of health and wellbeing in specific contexts, few studies have attempted to explore and confirm or refute these relationships on large scales. Cities, for example, may be appropriate units of analysis because they “represent discrete entities and are social and physical ecosystems in which exposure to green space may be comparatively limited but particularly valuable” [37] (p. 160). Furthermore, planning and management decisions are often made at the municipal level, enhancing the practical implications of city-level inquiries. Some studies even suggest that observed health benefits of green space for individuals may be even more pronounced when aggregated at larger community or city scales [38].

The few studies attempting to examine the relationship between health metrics and green space at the city level have produced mixed results. Using secondary data for U.S. cities, West et al. [68] demonstrated that urban park density was correlated with physical activity and healthy weight. Conversely, in their study of U.S. cities, Richardson et al. [37] found no associations between urban “greenness” and mortality from heart disease, diabetes, or lung cancer. Both of these studies focused explicitly on physical health, however, and did not account for more comprehensive measures of wellbeing. Florida et al. [22] examined socio-demographic correlates of subjective wellbeing in U.S. metropolitan areas, but their analysis did not account for the contributions of parks and green space.

Considering these variable findings and the increasing emphasis on urban health promotion, there is a growing need to understand the complex relationship between urban green space and human health and wellbeing at the city level [69, 70]. The contributions of public parks, specifically, warrant more attention. Research has shown that the type and quantity of green space matters when it comes to health outcomes, and structured or actively managed settings such as parks may generate greater benefits than unstructured natural areas [48, 71]. In fact, the Centers for Disease Control and Prevention has specifically identified parks as a key community feature that influences health promotion [72]. Furthermore, parks represent discrete management units that can be adapted and altered through design, maintenance and programming to promote specific health outcomes and policy responses that enhance residents’ wellbeing. The broader category of urban green space, on the other hand, generally spans multiple jurisdictions and is much more difficult to manage in coordinated fashion.

We sought to expand the literature on this topic by examining the specific relationship between urban park quantity, quality, and accessibility and citizens’ self-reported wellbeing at the city level, simultaneously accounting for the other previously specified variables (e.g., socio-economic factors, social factors) typically viewed as wellbeing correlates. We hypothesized that indicators of park quantity, quality, and accessibility, would emerge as significant explanatory variables in models predicting wellbeing at the city level.

## Methods

Our analysis utilized municipal and metropolitan statistical area-level (MSA) data for most of the largest cities across the United States. Data were obtained from a variety of secondary data sources that typically contained information for more than 100 U.S. cities (e.g., Gallup, Trust for Public Land, U.S. Census Bureau). However, information related to all of our dependent, independent, and covariate variables of interest–particularly data on park accessibility—were not available for all cities. After deleting records with missing data on one or more of the variables in our model, our effective sample size for analysis was reduced to 44 cities (S1 Table). This sample included most of the largest U.S. metropolitan areas, ranging in size from New York, NY (2010 population = 8,175,136) to Wichita, KS (2010 population = 382,373).

### Dependent Variable: Wellbeing

The dependent variable in our analysis was subjective wellbeing measured using the Gallup-Healthways Well-being Index (WBI), which has been described as “the most proven, mature and comprehensive measure of well-being in the world” (http://www.well-beingindex.com/about). Scale development, instrument refinement, and construct validation are based on decades of data collection in over 150 countries [12]. The latest version of the Gallup-Healthways WBI includes an overall index score based on five essential elements [19]: (1) Physical wellbeing refers to having good health and enough energy to get things done on a daily basis (it encompasses both physical and mental health domains that are sustained through healthy living habits); (2) Community wellbeing describes the sense of engagement individuals have with the area where they live (it refers to feelings of safety, security, and sense of local pride); (3) Social wellbeing pertains to the quality of relationships and love in life, accounting for the interactions and social connections that make life more enjoyable; (4) Financial wellbeing describes one’s economic life by fostering a sense of economic security and an ability to fulfill essential needs; (5) Purpose and associated career wellbeing center on how one occupies his/her time and enjoys what he/she does every day (it fosters enthusiasm about the future and a sense of self-worth that leads to fulfillment). Although the WBI is designed to produce a composite well-being score ranging from 0 to 100, it also produces individual scores for each of the five distinct elements of wellbeing that can be independently analyzed.

In the United States, the WBI data are collected through daily telephone surveys of adults, with at least 500 surveys occurring on about 350 days each year [73]. The U.S. aggregate wellbeing estimates are therefore derived from more than 160,000 independent telephone surveys, with specific sample sizes for each urban area varying based on city population. The dual-frame sampling includes random-digit-dial landline and wireless samples stratified to ensure adequate coverage across the country. Data were weighted to compensate for disproportionalities in demographic attributes, selection probabilities, and nonresponse rates. We used the 2014 MSA-level WBI data in our analysis (Table 1). These data are accessible via a paid subscription to the Gallup Analytics portal (https://analytics.gallup.com/).

**Table 1:** Variables Used in the Linear Regression Model Examining Factors Associated with Wellbeing in U.S. Cities.

Variable	Source	Mean	Median	Range (with cities)
**WBI-Overall** (Gallup-Healthways Wellbeing Index)	Gallup 2014	61.72	61.84	59.36 (Indianapolis, IN)– 63.61 (Raleigh, NC)
**WBI-Physical**	Gallup 2014	6.14	6.14	5.80 (Indianapolis, IN)– 6.36 (Los Angeles, CA)
**WBI-Social**	Gallup 2014	6.09	6.10	5.88 (Indianapolis, IN)– 6.29 (Raleigh, NC)
**WBI-Community**	Gallup 2014	6.08	6.10	5.72 (Detroit, MI)– 6.47 (Austin, TX; Raleigh, NC)
**WBI-Financial**	Gallup 2014	5.95	5.93	5.57 (Memphis, TN)– 6.45 (San Jose, CA)
**WBI-Purpose**	Gallup 2014	6.03	6.02	5.79 (Columbus, OH)– 6.50 (El Paso, TX)
**ParkPercent** (Percentage of total city area covered by parks)	TPL 2014	10.7%	9.0%	2.0 (Fresno, CA; Tucson, AZ) - 23.0% (San Diego, CA)
**ParkSpending** (Annual per capita spending on parks)	TPL 2014	$94	$76	$11 (Detroit, MI)—$250 (Washington, DC)
**ParkAccess** (Percentage of population within ½ mile of a park)	TPL 2014	63.5%	62.6%	26.5 (Charlotte, NC) - 98.2% (San Francisco, CA)
**NaturalAmenities** (Score on natural amenities scale that accounts for 6 variables related to climate and geography)	USFS 1999	1.84	0.33	-2.51 (Indianapolis, IN)– 10.52 (San Francisco, CA)
**SinglePercent** (Percentage of single individuals age 15 or older)	ACS 2014; MPI 2014	51.9%	52.2%	46.3% (Colorado Springs, CO)– 57.7% (Memphis, TN)
**LogIncome** (Log of median household income)	US Census 2010	10.76 ($48,108)	10.75 ($46,686)	$26,212 (Cleveland, OH)—$81,829 (San Jose, CA)
**WorkFulltime** (Percentage of adults employed full time)	Gallup 2013	46.2%	47.1%	38.9% (Sacramento, CA)– 54.4% (San Jose, CA)
**CollegeDegree** (Percentage of population with a Bachelor’s degree or higher)	US Census 2010	32.5%	30.0%	12.7% (Detroit, MI)– 57.4% (Seattle, WA)
**PopDensity** (People per Hectcre)	US Census 2010	19.8	13.5	3.7 (Oklahoma City, OK)– 92.5 (New York, NY)
**PopChange** (Percent population change from 2010–2012)	US Census 2012	2.5%	2.6%	-1.7% (Detroit, MI)– 6.6% (Austin, TX)

### Independent Variables: Park Quality, Quantity, and Accessibility

We used proxy variables to evaluate three aspects of public parks that research indicates are important predictors of wellbeing: quantity, quality, and accessibility [48, 49, 74]. Specifically, we used metrics from the Trust of Public Land’s (TPL) Park Score Index, which annually reports key metrics and rankings for the parks systems of the nation’s largest cities [75]. To estimate park quantity (ParkPercent), we added all acres of parkland managed by any public park agency (federal, state or municipal) within the boundaries of the municipality and divided this number by the total area of each municipality. To estimate park quality (ParkSpending), we used TPL’s approximations of annual, cost-of-living adjusted, per capita spending on parks, which included aggregate operating and capital expenditures for all public park agencies operating within a municipality. To facilitate interpretation of model coefficients, ParkSpending was converted from dollars per person to tens of dollars per person. To estimate park accessibility (ParkAccess), we used TPL’s calculation of the overall percentage of the city population within ½ mile, or 10-minute walking distance, of a park that can be reached via road networks unobstructed by obstacles (e.g., freeways, rivers, fences). All TPL data obtained through the 2014 City Park Facts report and online public databases (accessible at http://parkscore.tpl.org/) were aggregated to the city level (Table 1).

### Covariates: Other Correlates of Wellbeing

Although park-related variables were the focus of this analysis, their independent influence on wellbeing could not be accurately assessed without considering other potential correlates that have been identified in the literature [22]. To account for the potential influence of other environmental factors such as climate and geography, we used data from the U.S.D.A. Economic Research Service’s Natural Amenities Scale (http://www.ers.usda.gov/data-products/natural-amenities-scale.aspx), a composite index that incorporates the normalized contributions of six different meteorological and topographical variables including temperature, hours of sunlight, humidity, topography, and bodies of water for each county of the United States [76]. The scale was developed in 1999 and has not been updated since; however, it is unlikely that relative differences in these geographical and meteorological variables have shifted substantially in the past 15 years. Because natural amenities data were only available at the county level, we utilized scores for the most populous county within each MSA (Table 1).

To approximate income at the city level, we used estimates of 2010 median household income published by the U.S. Census Bureau (www.census.gov/quickfacts/) [77]. Following recommendations of other authors [21], we used the logarithm of estimated income in our model to emphasize relative, not absolute, differences among variable income levels (Table 1). To approximate education at the city level, we used 2010 estimates of the percentage of the adult population (age 25 or older) in each city that held a Bachelor’s degree [77]. To account for employment at the municipal level, we used estimates of the population to payroll rate, or the percentage of the population employed by an employer for at least 30 hours per week. These data were collected as part of Gallup’s regular survey effort, and we accessed the 2013 MSA-level data (2014 data were not available at the time of analysis) via the Gallup Analytics Portal (https://analytics.gallup.com/) (Table 1). To account for the complex influence of social relationships on wellbeing, we used a coarse approximation: the percentage of the overall adult population (age 15 or older) of each city that is single. Although this statistic is not a perfect proxy and likely varies along with other demographic correlates (e.g., age), it does indirectly integrate many of the key social variables believed to moderate the relationship between urban green space and wellbeing (e.g., lifestyle traits, household characteristics, living context) [70]. The 2014 data, obtained from the Martin Prosperity Institute [78], were based on three-year rolling averages of the single population percentage derived from the American Community Survey [79] (Table 1).

We also included two additional variables related to city population dynamics. To control for population size in the analysis, we included population density, measured as people per hectare and calculated using 2010 data from the U.S. Census Bureau (www.census.gov/quickfacts/) [77]. We also wanted to include some measure of population trends in each urban area, which tend to be a good indicator of social and economic trajectories. Population change estimates can be used to compare wellbeing scores for cities that are thriving (e.g., Austin, TX) versus those that are struggling (e.g., Detroit, MI). We calculated population changes from 2010–2012 using data from the U.S. Census Bureau [77] (Table 1). All of the data used in these analyses are available as supplementary online material (S1 Table).

### Data Analysis

We first assessed the binary relationships between each of the independent park variables (i.e., ParkPercent, ParkSpending, ParkAccess) and wellbeing (including overall WBI score and the scores for specific subdomains) using Pearson correlations. Given the small sample size, we used the bootstrapping method to estimate the accuracy of our sample correlation estimates. Specifically, we randomly sampled from our sample data 1,000 times to produce a population estimate of Pearson’s correlation coefficients, upper and lower limits of the 95% confidence intervals, and statistical significance.

After examining binary relationships, we used an information theory approach to examine a suite of eight potential OLS regression models including various combinations of correlates to identify the most parsimonious model with the best predictive power for overall wellbeing and each of the specific subdomains [80]. The candidate set of models examined for each dependent variable ranged in size from 3–10 predictors and included: (a) a model containing only the three park variables; (b) a model containing the three park variables and the Natural Amenities Scale; (c) two models containing different combinations of socio-demographic variables (with likely sources of collinearity removed); (d) one model containing four socio-demographic variables and the Natural Amenities Scale; and (e) three models containing different combinations of park variables, socio-demographic variables, and the Natural Amenities Scale, including a full model with all ten predictors. Because of the small sample size, we used the second order Akaike’s Information Criterion (AICc) and corresponding Akaike’s relative likelihood weights to identify the best models given the data structure [81]. We defined the confidence set of candidate models for each dependent variable as all models with Akaike weights within 10% of the highest value [82]. We elected to use only the top models in these confidence sets when evaluating parameter estimates for specific independent variables. By examining these top models and their corresponding effect sizes (measured by adjusted R^2^) and parameter estimates, we were able to determine the relative influence of the park variables and other potential correlates on wellbeing.

Prior to running the models, preliminary analyses were conducted to ensure that the assumptions required for OLS regression analysis were not violated. Because of anticipated relationships among many of the independent variables, multicollinearity was a concern–particularly for the full model including all 10 predictors. For example, CollegeDegree and LogIncome were highly correlated (*r* = 0.769, p <0.001). Nevertheless, their variance inflation (VIF) scores were less than 6.0, and the variables were retained because they both served as key controls (i.e., covariates). Correlations between the primary independent variables of interest, ParkPercent and ParkSpending (*r* = 0.356, *p* = 0.018), ParkPercent and ParkAccess (*r* = 0.421, *p* = 0.004), and ParkSpending and ParkAccess (*r* = 0.595, *p* = 0.001) were also significant, but their VIF values of 3.7 or lower were all within the acceptable limits for OLS regression. Potential issues of multicollinearity were minimized when interpreting the best models, however, because none of these models included more than eight predictors. Measures of standardized residuals (between -2.0 and 2.0) and Cook’s distance (< 0.16) were within acceptable ranges, suggesting that outliers were not a concern. Examination of residuals scatterplots confirmed that the assumptions of normality, linearity, and homoscedasticity were also satisfied [83]. The same preliminary analyses were conducted before interpreting all of the regression models.

## Results

Initial assessments using Pearson correlations suggested that percentage of parkland in each city was significantly associated with overall wellbeing at the city level (*r* = 0.496, *p* = 0.001). Per capita park spending and park accessibility were positively correlated, though not significantly, with wellbeing. All of the park variables were significant correlates of the physical and financial wellbeing subscales (Table 2). Cities with a greater percentage of land in parks also displayed higher average scores on the community wellbeing subscale (*r* = 0.340, *p* = 0.005). The only observed negative association was between park accessibility and the purpose subscale (*r* = -0.315, *p* = 0.030).

**Table 2:** Pearson Correlation Coefficients Examining Associations between Park Variables and Wellbeing Indicators for U.S. Cities (n = 44).

	ParkPercent		ParkSpending		ParkAccess	
**Variable**	*r*	95% *CI*^a^	*r*	95% *CI*^a^	*r*	95% *CI*^a^
WBI-Overall	0.496**	0.279–0.637	0.172	-0.095, 0.488	0.129	-0.131, 0.427
WBI-Physical	0.503**	0.241–0.702	0.278*	0.052, 0.548	0.342*	0.150, 0.588
WBI-Community	0.340*	0.076–0.544	0.059	-0.212, 0.355	-0.084	-0.346, 0.187
WBI-Social	0.261	-0.025–0.542	0.217	-0.071, 0.455	0.217	-0.081, 0.477
WBI-Financial	0.461**	0.222–0.707	0.415**	0.105, 0.660	0.424**	0.189, 0.631
WBI-Purpose	0.123	-0.252–0.285	-0.281	-0.531, 0.058	-0.316*	-0.552, 0.064

* and ** denote significance of correlation coefficient estimate at α = 0.05 and 0.01, respectively, following bootstrapping with 1,000 iterations

^a^95% confidence interval based on bootstrapping estimates with 1,000 iterations

Our information theory approach used AICc to compare eight candidate models and identify the best model for predicting overall WBI scores at the city level (Table 3). The best fitting regression model, with a likelihood of weight of 0.86, included six variables (PercentParks, NaturalAmenities, PopChange, LogIncome, SinglePercent, and WorkFulltime). This model explained almost 60% of the WBI score variance. The combination of variables in this model suggest that both environmental (e.g., park-related) and socio-demographic variables combine to influence overall wellbeing. An examination of regression parameter estimates for this best fitting model showed that, controlling for other correlates, cities with more park coverage had higher average overall WBI scores (*B* = 9.10, *p* = 0.001) (Table 4). Other significant correlates of overall WBI were the city’s natural amenities score (*B* = 0.14, *p* = 0.014), the percentage of the population that is single (*B* = -15.39, *p* = 0.009), the logarithm of median household income (*B* = -1.66, *p* = 0.044), and the two-year trend in city population (*B* = 0.29, *p* = 0.001).

**Table 3:** Model Selection Overview for Variables Associated with Overall Wellbeing Scores in U.S. Cities (n = 44).

Model (Parameters Included)	*K*^a^	*RSS*^b^	*AICc*	*ΔAICc*	*w*_*i*_^c^	Adj. *R*^*2*^
1. PopChange + LogIncome + SinglePercent + WorkFullTime + NaturalAmenities + PercentParks	6	17.99	-23.08	0	0.860	0.584
2. PopChange + LogIncome + SinglePercent + WorkFulltime + NaturalAmenities + PercentParks + ParkSpending + ParkAccess	8	17.16	-19.31	3.77	0.131	0.580
3. PopChange + PopDensity + LogIncome + CollegeDegree + SinglePercent + WorkFulltime + NaturalAmenities + PercentParks + ParkSpending + ParkAccess	10	16.96	-13.28	9.80	0.006	0.560
4. PopChange + LogIncome + PercentSingle + WorkFulltime + NaturalAmenities	5	24.84	-11.58	11.50	0.002	0.440
5. PopChange + LogIncome + PercentSingle + WorkFulltime	4	29.10	-7.17	15.91	<0.001	0.361
6. PopChange + PopDensity + LogIncome + PercentSingle + WorkFulltime + CollegeDegree	6	27.76	-3.96	19.12	<0.001	0.357
7. NaturalAmenities + ParkPercent + ParkSpending + ParkAccess	4	31.67	-3.44	19.64	<0.001	0.304
8. ParkPercent + ParkSpending + ParkAccess	3	37.34	1.38	24.46	<0.001	0.200

^a^*K* = number of parameters in the model

^b^*RSS* = Residual Sum of Squares

^c^w_i_ = Akaike weights (relative likelihoods) for Model *i*

**Table 4:** Parameter Estimates for Best-fitting OLS Regression Model^a^ Depicting Factors Associated with Overall Wellbeing Index Scores for U.S. Cities (n = 44).

Variable	*B*	*SE*	*β*	Sig.
Constant	89.21	9.43		
ParkPercent	9.10	2.43	0.457	0.001
NaturalAmenities	0.14	0.06	0.438	0.014
PercentSingle	-15.39	5.60	-0.364	0.009
LogIncome	-1.66	0.80	-0.355	0.044
WorkFulltime	-1.28	5.16	-0.046	-0.248
PopChange	0.29	0.08	0.480	0.001

^a^Overall WBI model fit statistics: *F*(6,37) = 11.04, *p* < 0.001, Adj. *R*^*2*^ = 0.584

Using the AICc comparison approach and the same set of candidate models, we also identified the best fitting models for each of the 5 subdomains of wellbeing (Table 5). In almost every case, the best fitting model was obvious. The best regression model for physical wellbeing (Akaike *w*_*i*_ = 0.422) included the three park variables and natural amenities and explained 54% of the score variance. ParkPercent (*B* = 0.64, *p* = 0.045) and NaturalAmenities (*B* = 0.02, *p* < 0.001) were the only significant predictors in this model, though ParkAccess and ParkSpending were also positively correlated with physical wellbeing.

**Table 5:** Top Two Models Predicting each Subdomain of Wellbeing in U.S. Cities (n = 44).

Outcome Variables & Models	*K*^a^	*RSS*^b^	*AICc*	*ΔAICc*	*w*_*i*_^c^	Adj. *R*^*2*^
**Physical Wellbeing**						
1. NaturalAmenities + ParkPercent + ParkSpending + ParkAccess	4	0.351	-201.55	0	0.422	0.535
2. PopChange + LogIncome + SinglePercent + WorkFulltime + NaturalAmenities + ParkPercent + ParkSpending + ParkAccess	8	0.275	-201.19	0.35	0.354	0.593
**Community Wellbeing**						
1. PopChange + LogIncome + SinglePercent + WorkFulltime + NaturalAmenities + PercentParks	6	0.523	-178.75	0	0.892	0.663
2. PopChange + LogIncome + SinglePercent + WorkFulltime + NaturalAmenities + ParkPercent + ParkSpending + ParkAccess	8	0.512	-173.85	4.91	0.077	0.651
**Financial Wellbeing**						
1. PopChange + LogIncome + SinglePercent + WorkFulltime + NaturalAmenities + ParkPercent + ParkSpending + ParkAccess	8	0.539	-171.58	0	0.360	0.510
2. PopChange + LogIncome + SinglePercent + WorkFulltime + NaturalAmenities + PercentParks	6	0.616	-171.55	0.03	0.354	0.470
**Purpose**						
1. PopChange + LogIncome + SinglePercent + WorkFulltime + NaturalAmenities + PercentParks	6	0.480	-182.53	0	0.381	0.350
2. PopChange + LogIncome + SinglePercent + WorkFulltime	4	0.544	-182.27	0.26	0.334	0.301
**Social Wellbeing**						
1. ParkPercent + ParkSpending + ParkAccess	3	0.350	-204.70	0	0.590	0.021
2. NaturalAmenities + ParkPercent + ParkSpending + ParkAccess	4	0.347	-202.05	2.05	0.212	0.004

^a^*K* = number of parameters in the model

^b^*RSS* = Residual Sum of Squares

^c^w_i_ = Akaike weights (relative likelihoods) for Model *i*

The best regression model for community wellbeing (Akaike *w*_*i*_ = 0.892) included six environmental and socio-demographic variables and explained 66% of the score variance (Table 5). ParkPercent (*B* = 1.41, *p* = 0.002) was a significant positive predictor of community wellbeing. Other significant predictors in this model were SinglePercent (*B* = -5.51, *p* < 0.001), PopChange (*B* = 0.06, *p* < 0.001), and LogIncome (*B* = -0.38, *p* = 0.008).

Financial wellbeing was the only domain whose confidence set included more than one model, though variables in both these models were very similar. The best regression model for financial wellbeing (Akaike *w*_i_ = 0.360) included eight environmental and socio-demographic variables and explained 51% of the score variance (Table 5). All of the park variables were included in this regression model. Although each of these park variables was positively associated with financial wellbeing, none of these associations were statistically significant. In fact, the only significant predictors of financial wellbeing in the best-fitting model were WorkFulltime (*B* = 2.08, *p* = 0.039) and SinglePercent (*B* = -2.55, *p* = 0.020).

The best regression model for the purpose domain (Akaike *w*_*i*_ = 0.381) included six environmental and socio-demographic variables and explained 35% of the score variance. ParkPercent (*B* = 0.83, *p* = 0.043) was a significant positive predictor of purpose. Other significant predictors in this model were PopChange (*B* = 0.05, *p* < 0.001), WorkFulltime (*B* = -1.94, *p* = 0.027), LogIncome (*B* = -0.32, *p* = 0.017), and SinglePercent (*B* = -2.37, *p* = 0.014).

None of the models in the candidate set explained social wellbeing with any degree of predictive power (Table 5). The best regression model (Akaike *w*_*i*_ = 0.590) included the three park variables, but explained only 2% of the score variance. Socio-demographic variables were entirely absent in this model. Although each of the park variables was positively associated with social wellbeing, none of these associations were statistically significant.

## Discussion

Our study is among the first to investigate the relationship between urban parks and subjective wellbeing at the city level using one of the most comprehensive and extensively validated metrics available globally, the Gallup-Healthways Wellbeing Index [19]. The information theory approach we applied revealed that residents reported higher levels of subjective wellbeing in cities with a greater quantity of park coverage (the proportion of total city area reserved as public parks), even after controlling for a range of other potential geographical and socio-economic correlates. This finding is supported by an array of other studies that demonstrate the multiple ways that green infrastructure, generally [36, 41, 67, 84], and public parks, specifically [48, 49], contribute to public health.

Weaker relationships were observed for the variables we used to approximate park quality (per capita spending on parks) and accessibility (the percentage of the population within ½ mile of a park) at the municipal level. Although both were significantly associated with physical and financial wellbeing in the correlational analysis, neither emerged as a significant predictor in any of the multivariate regression models. While the hypothesized link between park coverage and wellbeing was confirmed, the absence of association for the other two park variables was somewhat unexpected.

Many studies have shown that the health-related benefits parks provide are linked to specific indicators of park quality such as facilities, activities, and programming [69, 85–87]. Perhaps per capita agency spending on operating costs and capital improvements was not a sufficient proxy for these aspects of park quality. Future studies could attempt to characterize the quality of a municipal park system using a more diverse set of indicators. The absence of a relationship between our measure of park accessibility and wellbeing was also a surprise. However, even if a population has access to parks, park use may still be constrained by other local factors such as perceptions of safety and accessibility, park amenities/programs, and nearby environmental hazards. Although some research highlights positive relationships between urban park proximity, perceived health [39], and physical activity [88], other studies have revealed somewhat inconclusive results [89]. These incongruous findings often reflect differences in scale, with potential effects masked when analyses are conducted at the city (vs. neighborhood) level. Such results, coupled with our present analysis, highlight the importance of examining associations between the spatial distribution of urban parks and residents’ wellbeing at multiple scales.

### Public Parks and Wellbeing

The strong positive relationship we observed between urban park coverage and physical wellbeing, or having good health and enough energy to get things done on a daily basis, was anticipated based on previous research. Contributions of public parks to physical activity in urban settings are widely documented at both the neighborhood [48, 86, 90] and city level [49], and many studies have demonstrated that parks and urban green space provide additional indirect physical health benefits via ecosystem services such as air and water purification [40]. Furthermore, the WBI measure of physical wellbeing also incorporated elements of mental and psychological health, both of which appear to be linked to direct and indirect contact with nature in urban settings [9, 38, 55–57]. In short, our U.S. city-level analysis supports a growing body of evidence highlighting significant associations between parks and physical health and wellbeing.

Urban park coverage was also positively associated with community wellbeing, or the sense of engagement individuals have with the place where they live. By providing a unique form of social gathering space in congested cities, parks and other natural settings facilitate social interactions and collaboration [63] and contribute to a sense of community or neighborhood attachment [62, 64–66, 91]. Parks may therefore help to facilitate the construction of social capital and subsequent perceptions of community wellbeing that are defining attributes of many sustainable urban communities. This supposition was supported by the best fitting model for social wellbeing, which included all three of the park variables yet none of the other potential socio-demographic correlates. However, it should be reiterated that all of our models for social wellbeing displayed poor predictive power. Because the WBI measures of social wellbeing focus primarily on personal relationships and not the context in which they evolve, social aspects of wellbeing might be more strongly influenced by individual-level variables not considered in the present analysis.

A significant positive relationship was also observed between park coverage and residents’ sense of purpose, broadly defined as liking what you do every day and being motivated to achieve goals. Sense of purpose is highly individualistic, often dictated by personal circumstances and actions far removed from physical settings (e.g., parks). However, it is possible that, by providing a space for recreation, reflection, and cognitive growth, parks can supply urban residents with a sense of satisfaction and goal fulfillment that fosters sense of purpose [9]. Parks may also indirectly foster a sense of purpose by contributing to other components of subjective wellbeing, generating a system of synergistic interactions that collectively enhance all aspects of wellbeing. However, empirical documentation to support these suppositions is lacking. Alternatively, observed correlations could simply be a byproduct of other relationships. For example, cities with more parks might boast a higher tax base and more affluent residents that tend to be distracted by technology, media, and a fast-paced professional world, diluting (or enhancing) their core sense of purpose in life.

Our correlational analyses showed that park quantity, quality, and accessibility were all positively associated with financial wellbeing, a measure reflecting an individuals’ sense of economic security and the ability to fulfill essential needs. All of the park variables were also present in the best fitting regression model for financial wellbeing, but the statistical significance of these relationships vanished after isolating the contributions of parks by controlling for socio-economic variables (including income and employment status). However, other empirical evidence supports a potential link between parks and financial wellbeing. Studies show that urban property values often increase near parks and public green space [92, 93], and areas with more parks are therefore more likely to attract higher-income residents. The expansion of urban park networks and associated programming may also create new employment opportunities for urban residents. Even if parks do not contribute directly to financial wellbeing, they often represent a common attribute of prosperous urban communities where financial security and economic opportunities abound.

### Other Correlates of Wellbeing

Although our study was designed to investigate relationships between public parks and wellbeing in U.S. cities, analyses also highlighted significant relationships between wellbeing and several other covariates. Scores on the Natural Amenities Scale (which accounted for variables such as temperature, sunny days, topography, and proximity to water) were significant positive correlates of overall wellbeing and physical wellbeing, specifically. This finding supports research showing that climatic and geographic variables have a highly significant effect on country-wide self-reported levels of happiness [30, 32]. It also underscores the powerful influence of the natural environment on human health and wellbeing. The percentage of the adult population employed full time was a significant positive correlate of financial wellbeing, as would be expected with the income that comes with a full time job, yet this variable was inversely related to sense of purpose. The percentage of the city population over age 15 and single was negatively associated with overall wellbeing, community wellbeing, financial wellbeing, and sense of purpose, supporting previous research suggesting partnered and married people typically are happier than their single counterparts [94]. Surprisingly, median income was negatively associated with overall wellbeing, community wellbeing, and sense of purpose. However, as previously mentioned, other researchers have noted that income alone may be a poor predictor of subjective well-being [20, 21]. The population trajectory in cities from 2010–2012 was a significant positive correlate of overall wellbeing and the community and purpose subdomains. This relationship helps to validate the assumption that cities whose residents report higher levels of wellbeing are growing and thriving, while cities with lower levels of wellbeing are not. Causality in this case is not clear, but the strength of association is difficult to dispute. To expand predictive power, future models assessing wellbeing at the city level could also attempt to account for individual differences in personality traits, relationships, and other factors that have been shown to be important correlates of subjective wellbeing and happiness on smaller scales [95].

### Limitations

Several limitations of the present analysis should be noted. First, inferences may be constrained by variation in temporal and spatial scales of measurement across the various data sources. For example, aggregate wellbeing data from the WBI were reported at the MSA level, while other metrics were reported at the incorporated city (e.g., TPL and Census data) and county (e.g., Natural Amenities Scale data) level. Though such inconsistencies can be problematic for city-level analyses [37], conversion to a standardized geographical unit encompassing an identical sample population was not possible given the different data sources, collection methodologies, and reporting scales. The size of our study sample was also limited by incomplete data coverage across U.S. urban areas. Although WBI data were available for over 180 urban areas, standardized park data (and park accessibility data, specifically) were only available for approximately 50 cities, reducing the effective sample size used in the analysis. Our work therefore demonstrates a need for more consistent and complete city-level data across temporal and spatial scales. It should also be noted that our coarse analysis at the MSA or city level does not account for attributes of specific urban neighborhoods, which may have more nuanced (and perhaps more significant) impacts on the health and wellbeing of individual residents. We were most interested in national-level patterns, but site-based research can and has yielded additional insights regarding the specific relationships between parks and wellbeing that can inform park management and health promotion on smaller scales [48]. Findings reported here may differ in smaller communities and other more localized contexts. Finally, it is important to note that the relationships depicted in these models indicate strong associations, but not necessarily causation. Time-ordered, experimental approaches are needed to support theoretical assumptions and establish more concrete causal links between the various domains of wellbeing and its significant correlates (including variables related to parks). Despite these limitations, our study provides a preliminary look at the relationship between urban parks and aggregate well-being on a scale that has rarely been explored, highlight broader implications for urban planning and management.

### Parks and Wellbeing: Implications for the Future

As national initiatives such as Healthy Parks, Healthy People and the Park Prescription movement gain momentum [96], researchers and practitioners are constantly searching for empirical evidence to provide additional insights regarding the relationship between urban parks, green space, and population health. Our exploratory study revealed significant associations between public park coverage and subjective wellbeing–particularly the physical and community wellbeing–of residents in U.S. cities. These findings highlight the important contributions of park networks to multiple aspects of sustainable urban planning and design [97] and demonstrate positive park-related externalities that are often underrated in urban economics and policy [98]. Our analysis, however, did not consider the specific use and non-use values of parks, including the way that urban parks are both perceived and experienced [99]. Future inquiries could therefore consider the different ways that individual urbanites come to know, perceive, and interact with parks and green space, and the health implications of these perceptions and interactions [9, 100]. For example, is park provision alone adequate, or is a particular dose of nature needed to generate tangible results [74]? Through which pathways can parks precipitate specific health outcomes [101]? To expand the present body of work and explore causal relationships, more experimentation and targeted interventions are needed to assess the impacts of parks on different components of health and wellbeing and develop a more mechanistic understanding of these relationships [42, 69, 101].

The municipal-level variables we examined only accounted for broad park coverage, not the distributional equity of those park resources across urban landscapes. This is a common limitation in city-level analyses, but it might have significant implications for policy and practice. Studies have highlighted racial/ethnic and socioeconomic disparities in park quality/funding [102, 103] and accessibility [104–106]. Since parks and urban green spaces provide ecosystem services that can buffer many urban health problems in high-risk populations [107–109], additional analyses of the park-wellbeing relationship across specific socio-demographic blocks is needed to generate equitable and informed management decisions that reflect the interests of diverse constituents.

Although parks represent discrete units that are easier to manage for particular outcomes, they are not the only type of green space contributing to the wellbeing of city residents. Other studies using spatial analytical techniques (e.g., canopy cover) have shown that general urban green space, not simply confined to parks, confers a variety of physical and mental health benefits [39, 46, 55]. For example, research demonstrates how both urban greenways [110] and community gardens [53] positively impact the health and wellbeing of urban residents. Future studies could expand the concept of parks and green space to encompass all types of urban ecosystem services and the benefits they provide to people [8, 45, 109, 111], ultimately assessing their impacts on health, happiness, and urban quality of life.

## Supporting Information

S1 Table Spreadsheet Containing Data Used to Examine Wellbeing Correlates in 44 U.S. Cities. (XLSX)
